# Continuing surgical education of non-technical skills

**DOI:** 10.1016/j.amsu.2020.07.062

**Published:** 2020-08-08

**Authors:** Masaomi Yamane, Seiichiro Sugimoto, Etsuji Suzuki, Keiju Aokage, Mikio Okazaki, Junichi Soh, Makio Hayama, Yuji Hirami, Takashi Yorifuji, Shinichi Toyooka

**Affiliations:** a Departments of General Thoracic Surgery and Breast and Endocrinological Surgery, Okayama University Graduate School of Medicine, Dentistry and Pharmaceutical Sciences, Okayama, Japan; b Department of Epidemiology, Okayama University Graduate School of Medicine, Dentistry and Pharmaceutical Sciences, Okayama, Japan; c Department of Thoracic Surgery, National Cancer Center Hospital East, Kashiwa, Japan; d Division of Thoracic Surgery, Department of Surgery, Kindai University Faculty of Medicine, Osaka, Japan; e Department of Thoracic Surgery, Japanese Red Cross Okayama Hospital, Okayama, Japan; f Department of General Thoracic Surgery, National Hospital Organization Okayama Medical Center, Okayama, Japan

**Keywords:** Non-technical skills, Patient safety, Thoracic surgery

## Abstract

**Background:**

The non-technical skills for surgeons (NOTSS) system was developed as a tool to assess surgical skills for patient safety during surgery. This study aimed to develop a NOTSS-based training system for surgical trainees to acquire non-technical skills using a chest surgery scenario in a wet lab.

**Materials and methods:**

Trainees were categorized into three subgroups according to the years of experience as follows: Level A: 6 years or more; Level B: 3–5 years; and Level C: 1–2 years. Three stages of surgical procedure were designed: 1. chest wall resection and right upper lobe lobectomy, 2. right middle lobe sleeve lobectomy, and 3. right lower lobe lobectomy. One instructor was assigned to each operation table, who evaluated each participant's NOTSS scores consisting of 16 elements.

**Results:**

When comparing average NOTSS score of all the three procedures, significant differences were observed between Level A, B, and C trainees. As an example of varying elements by procedure, Level A trainees demonstrated differences in Situation Awareness, and a significant difference was observed in Level C trainees regarding the elements of Decision Making. On the contrary, no significant difference was observed among Level B trainees. In the comparison between first-time and experienced participants, a significant improvement was observed in some elements in Level B and C trainees.

**Conclusion:**

This study highlights the usefulness and feasibility of the NOTSS scoring system for surgeons with different experiences and the effectiveness of providing feedback to trainees during intraoperative handoffs in a wet lab.

## Highlights

• Team training was effective for trainees of different levels.
• Surgeons need continuing education of a non-technical skills.

## 1 Introduction

Despite significant effort to avoid it, human error is a major cause of medical accidents and adverse events, which could lead to life-threatening complications in the perioperative period. It has been reported that the occurrence of communication error and its association with critical situations are more significant than those of technical error during the surgical procedure [[1], [2], [3]]. Approximately half of the adverse events are due to poor non-technical skills in the surgical team [[1], [2], [3], [4], [5]]. Thus, it is important to observe and measure the non-technical intraoperative skills of surgeons, and provide structured feedback to improve patient safety.

Non-technical skills for surgeons (NOTSS), developed as a tool to assess surgical skills for patient safety during surgery, has gained global popularity [6,7]. It includes 4 important categories for surgeons to achieve the goal of a surgical team, situation awareness, decision making, communication and teamwork and leadership. However, it is difficult to routinely apply it as on-job training, especially in a clinical situation of thoracic surgery [[8], [9], [10]].

Currently, animal-based surgical training is widely practiced, providing surgeons with a near-clinical situation in the operating room (OR). Surgical training often focuses on device usage, commonly employed surgical techniques (endoscopic, single-port access and robotic-assisted surgeries) and some specialized procedures; however, the utility of NOTSS assessment has been underappreciated and should be emphasized as part of a comprehensive training program [7,8].

Accordingly, we developed a NOTSS-based training system for surgical trainees to acquire non-technical skills (by supplementing regular training) in a chest surgery mimicking clinical OR setting at a wet lab training facility. This study investigated the feasibility of the NOTSS scoring system, and how feedback improves the scores of trainees in this setting, focusing on potential experience-based differences in its effectiveness among the trainees.

## 2 Materials and Methods

### 2.1 Training seminar

The study analyzed data from evaluation sheets obtained from four training seminars in total, held annually from 2017 to 2020. There were 5–6 operating tables in each seminar, and the surgical team consisted of 3 members; therefore, the number of participants was 15 or 18. Pre and post seminar self-assessment tests, including non-technical skill taxonomy, were conducted for both participants and instructors. We have sent all Volunteers (surgical trainees and instructors) fully informed consent and were notified not to use the evaluation sheet if they declined consent (optout). The Okayama University Graduate School of Medicine, Dentistry and Pharmaceutical Sciences and Okayama University Hospital, Ethics Committee approved this study (approved on November 22, 2019, number 1908-003).

### 2.2 Participants

Trainees who desired to be surgeons or who had already participated or completed a surgical residency program and annually recruited participants to the training hospitals were eligible for the seminar. Trainees were categorized into three subgroups, depending on their level of experience as follows: Level A: 5 years or more; Level B: 3–5 years; and Level C: 1–2 years. The average years of experience in Levels A, B, and C were 6.70 years, 3.81 years, and 1.51 years, respectively. All participants provided informed consent prior to their inclusion in the study. The number of participants was 69 from 22 training hospitals [Table A1] in 10 prefectures and one country (China), and 204 evaluation sheets were available for this study.

### 2.3 Instructors

Instructors were selected from certified thoracic surgeons of various training hospitals. They were informed of their roles in the assessment of trainees using NOTSS scores (during surgery at seminars). All instructors met prior to the seminar to confirm the NOTSS criteria for evaluation, and to establish standard feedback for the trainees. Each operation table was assigned one instructor, who was responsible for evaluating the NOTSS score of each surgical trainee participant.

### 2.4 Surgical procedure

Large White pigs weighing approximately 45 kg were used in this study. General anesthesia (single lung ventilation) was introduced and maintained by a veterinarian and the condition and environment of the animals during surgery was controlled. Scheduled surgical procedures were designed as follows: 1. chest wall resection and right upper lobe lobectomy, 2. Right middle lobe sleeve lobectomy, and 3. Right lower lobe lobectomy (that is, completion pneumonectomy) [Table A2]. Surgical procedures were conducted in accordance with the ethical standards in the Declaration of Helsinki. The work has been reported in accordance with the ARRIVE guidelines (Animals in Research: Reporting In Vivo Experiments) [11]. UIN (Unique Identifying number) for this study, given by the Research Registry, is researchregistry56503 (https://www.researchregistry.com/browse-the-registry#home/registrationdetails/5ecfbc0098ff7600151fcaf2/). The animal protocols were approved by the animal committee in the institution.

### 2.5 Surgical team member rotation

One surgical team consisted of three participants (one from each level). The roles of members including the operator, a first assistant, and a second assistant (thoraco-scopist) were switched between procedures. One or two members of the surgical team were rotated in each procedure (Table A2). Staff of the same hospital were not assigned to the same team to minimize evaluation bias due to interpersonal relationship. Similarly, instructors and participants from the same institution were not paired together to avoid any personal factors that would confound the evaluation or feedback process. Following the completion of all procedures, the surgical teams presented a general report (audio and video) covering each surgical procedure.

### 2.6 Trainee performance evaluation using the NOTSS scoring system

The classification of NOTSS includes four categories as follows: Situational Awareness, Decision Making, Communication and Teamwork, and Leadership. Each category comprises three domains, and evaluates a total of 16 elements with four category ratings and 12 components. Each element is rated on a 4-point Likert scale (range from 1 to 4), with a higher score indicating better skill. A description of the criteria for evaluating each observed behavior is detailed in the NOTSS system manual. Instructors at each table (without scrubbing) evaluated all 3 members of the surgical team for each procedure according to the scale of NOTSS system. Therefore, each participant was evaluated three times, from procedure 1 to 3. Consequently, instructors would end up evaluating nine surgical trainee participants, in total. In the hand-off feedback, the surgery was stopped in any situation, and the trainee was provided feedback from the instructor according to the NOTSS evaluation sheet. The trainees, in turn, commented on “what could be done” and “what should have been done”. Following the completion of the procedures, the evaluation sheets were collected and analyzed.

### 2.7 Statistical analyses

Due to the small sample size, more conservative statistics were used, and comparisons were conducted using Wilcoxon rank sum test or Kruskal-Wallis test. Data were analyzed using the JMP pro software (SAS Institute Japan Ltd.). A p-value <0.05 (two-sided) was considered statistically significant.

## 3 Results

### 3.1 Surgical outcome

The instructors intervened in the procedures only on occasions of challenges such as bleeding that could become critical, team conflict, and disorientation of the anatomical structures, among others. With the exception of a case with time limitation, all procedures were successfully performed without animal death, and instructors needed to support the procedure in four cases (5.7%).

### 3.2 Changes in awareness of NOTSS after the seminar

Improvements were observed in all categories of trainees between pre- and post-seminar self-evaluation tests. The self-evaluation score of the instructor was higher than that of the participant; however, an increase was observed in the instructor's self-evaluation score following the seminar (almost full score, no data shown). Post-seminar self-evaluation tests similarly included free comments, and almost all feedbacks regarding the participation in this seminar by the instructors were positive.

### 3.3 NOTSS score differences between levels

Significant differences were observed between trainees in the three experience levels across all 16 evaluation elements [Table B]. Throughout the procedures, scores of the Level A trainees tended to exceed those of Level B and C trainees; however, not all elements were statistically different in each procedure. In Procedure 1, only 5 of the 16 elements reached statistically significant difference among Level A, B and C trainees: including “Selecting and communicating option” (p = 0.028), “Coordinating team activities” (p = 0.0041), “Leadership” (p = 0.0001), “Supporting others” (p = 0.0013) and “Coping with pressure” (p = 0.0045) In procedure 2, additional six elements (a total of 11 elements, including the elements from procedure 1) were observed to be significantly different between trainees of different experience levels. In procedure 3, all the evaluated elements significantly differed among the trainees at different levels.

### 3.4 Improvement in NOTSS scores during the training

As shown in Table C, we observed significant differences between the 3 procedures only in the following three elements: “Situation Awareness” (p = 0.006), “Gathering information” (p = 0.0005) and “Considering options” (p = 0.0136). In the first two element, the NOTSS scores gradually improved through the procedures, whereas a J-shaped pattern was observed for the “Considering option” element in trainees at all levels.

When examining the changes in NOTSS score within each Level, a significant improvement was observed in the following three elements for Level A trainees: “Situation Awareness” (p = 0.0049), “Gathering information” (p = 0.0233), and “Considering options” (p = 0.0354). In Level C trainees, a significant difference was observed in the scores of 2 elements: “Decision Making” (p = 0.0362), and “Establishing a shared understanding” (p = 0.0429). In addition, marginally significant differences were observed in the elements of “Selecting and communicating option” (p = 0.0569), and “Communication and teamwork” (p = 0.0622). Unlike Level A and C trainees, no significant differences were observed among Level B trainees across any procedure.

### 3.5 Differences in training experience at seminars

When comparing the first-time and two or more times participants, some elements significantly improved through procedures 1–3 in Levels B (4 of 16 elements) and C (10 elements); [Table D]. The improved elements mainly included those related to Decision Making and Communication and Teamwork in Level B trainees, and Situation Awareness also improved in Level C trainees. No significant improvement was observed in Level A.

## 4 Discussion

The NOTSS System Handbook (ver.1.2 released in 2012) was established by the University of Aberdeen, The Royal College of Surgeons of Edinburgh for structuring observation, rating and feedback of surgeons’ behaviors in OR. Jung et al. reviewed and critically appraised 12 literatures in which non-technical skills of surgeons were measured using this tool, and described that sensibility is assessed using ‘enlightened common sense’, a mixture of ordinary common sense and understanding of clinical reality [12].

In 2016, the Medical Accident Investigation Committee published a report, based on research and analysis from the Gunma University Hospital on medical accidents in Japan, reiterating the need for surgeons to acquire non-technical skills. NOTSS system training, particularly for the older generation of surgeons, was recently introduced in the workshop [13] or the seminar held by Japanese Association of Surgical Education.

This is the first study to examine the efficacy of the NOTSS system during chest surgery in a near-clinical OR setting, with a particular focus on how the receival of feedback based on the NOTSS handbook improves training outcomes. To maximize effectiveness of this program, we *a priori* intended to provide feedback to trainees during intraoperative hand-offs, which would generate a relatively safe atmosphere for the trainees and instructors to discuss, reflecting the skills, attitudes and plans during the operation. With this hands-on guided approach, the trainee can proceed to the next step with ideas for improving the procedure.

In a randomized controlled trial by Dedy et al. [14], the non-technical skills of senior general surgery residents were evaluated with the effect of coaching during laparoscopic cholecystectomy in a simulated OR. Gostlow et al. reported that NOTSS scores of the senior trainees were typically higher than those of the juniors in the study using a simulated scenario designed to challenge NOTSS [15]. Our results revealed a significant difference in NOTSS scores between junior and senior surgeons. Moreover, it was apparent that regardless of the years of experience, short-term training effects are more obvious in individuals whose roles inherently come with added responsibilities. Additionally, it was observed that maintaining the effects of training in the long-term can be more difficult for senior-level trainees. Overall, this tool is reasonable for improving to assess the non-technical skills of surgeons, and surgeon instructors should undoubtedly be recommended for use in trainee evaluation and feedback or coaching [15].

Among Level A trainees, significant differences were observed in the following three elements (through the three procedures); “Situation Awareness”, “Gathering information”, and “Considering option”. In this program, each team comprised unrelated surgeons, which was designed for situations that require effort to improve the quality of surgical performance. Because team building was, therefore, a very urgent objective at the commencement of each surgery, senior trainees may have been more motivated to take the initiative to establish a relationship in the team, focusing on the aforementioned three elements. Interestingly, “Gathering information” and “Understanding information” tended to steadily increase in Level C through the procedures, and their average scores in procedure 3 were close to the scores in Level B. It was *a posteriori* interpreted that, among younger surgeons, further information about procedures would drive the motivation to operate. In addition, it should be noted that Level B trainees might have felt intimidated to actively participate or interact with the instructors and other participants in the “middle” position of the teams.

It has been reported that a decrease in NOTSS scores, particularly of the leadership category, was observed in surgeons with an experience of 10–20 years [16]. It should be noted that trainees were younger, and a different training procedure was employed in this study; however, our findings suggest that there is a long-term retention of the skills learned in this program. This is encouraging and suggests that this training program could be useful in preventing the drop in NOTSS scores occasionally observed among more experienced surgeons [17,18].

One of the limitations of this study is that the actual operating performance of the participants was not evaluated following the seminar. In addition, the same NOTSS scoring system was used for all trainee levels as it focused on developing seminars to train different levels of surgical trainees, which may have led to unequal ratings. Although we intended to minimize the evaluation bias by designing the grouping of surgical trainees and paired instructors, future studies should improve level of inter-rater reliability by giving a thorough guide about rating to the instructors [12].

In the event some difficulties arise in a real clinical environment, these findings suggest the significance of providing appropriate feedback immediately before, during, or after the surgery for effective training of surgeons of varying experience, particularly younger surgeons. We considered that high-scoring level A trainees tended to be good coaches for junior trainees, but our analysis of the evaluation results did not show any difference. Assessing whether the combination of team members results in change, the quality of surgical progression, and the surgical skills themselves may be of interest in future studies.

## 5 Conclusions

A NOTSS-based training system was developed for acquiring non-technical skills (by supplementing normal training) in a chest surgery that simulates a clinical OR setting in a wet lab training facility. The findings demonstrate the usefulness and feasibility of the NOTSS scoring system and the intraoperative feedback (using results of NOTSS scoring) in providing surgical trainees with varying experience short-term or long-term impact in a wet lab.

## Provenance and peer review

Not commissioned, externally peer reviewed.

## Ethical approval

The Okayama University Graduate School of Medicine, Dentistry and Pharmaceutical Sciences and Okayama University Hospital, Ethics Committee approved this study (approved on November 22, 2019, number 1908-003).

## Sources of funding

No funding.

## Author contribution

Drs S.S., E.S., K.A., M.O., J.S., M.H., Y.H., T.Y. and S.T. contributed to study design and conception, and as instructors at a seminar and collected evaluation sheets. All authors contributed to the analysis and interpretation of data and manuscript drafting and approved the final version of the manuscript.

## Registration of research studies

researchregistry56503

https://www.researchregistry.com/browse-the-registry#home/registrationdetails/5ecfbc0098ff7600151fcaf2/

## Guarantor

Masaomi Yamane.

## Consent

We have sent all Volunteers (surgical trainees and instructors) fully informed consent and were notified not to use the evaluation sheet if they declined consent (opt out).

## Declaration of competing interest

Authors don’t have any conflict of interest.
